# Annotations

**Published:** 1907-03-09

**Authors:** 


### ANNOTATIONS.

### The Title of "Dr."

Every person practising medicine under a legal qualification is in the general view a "doctor," and to deny such a person the right to this title carries the implication that in some respect his professional position is defective. The functions, rights, and responsibilities of all qualified practitioners have one and the same legal basis, though to the layman differences in specific designations seem to imply differences in value and kind. Admission to the official register may take place through many portals, but once secured it carries the same consequences for all. There is thus something to be said for the proposal that all shall have the same descriptive name, and this the one which agrees with an almost universal custom. The University graduate would, of course, still enjoy his sole right to the use of his degree (M.D.), but no practitioner would be denied the use of the term which the public interprets as the equivalent of a legal qualification to practise. Possibly both those who advocate, and those who oppose this suggestion attach undue importance to it. In practice the public has a full opportunity to estimate the general worth and capacity of individual practitioners and technical details in regard to qualification are of essentially minor importance. Particular titles and designations will certainly not retain, even if they attract, confidence unless there is something more substantial to support them.

### Hæmaturia due to Bilharzia Hæmatobia.

In a paper on this subject, read by Mr. H. J. Curtis before the Medical Society on February 25, and in the discussion which followed, several points of interest are to be noted. First is a re-statement of the well-established truth that the sole evidence of the parasite may be the passage of blood in the urine. This needs to be borne in mind, otherwise a mysterious case of hæmaturia may fail to receive its adequate interpretation. The diagnosis is readily made, as the deposit from the urine when examined under the microscope at once reveals the characteristic ova. All this is of great practical moment to the practitioner in this country, for presumably as a result of the movements of troops and greater communication with South Africa generally cases of hæmatobia are certainly becoming more frequent in London. The same thing has been noticed in parts of Africa other than the east coast, where, as is well known, the disease is endemic. Thus the case recorded by Mr. Curtis was observed by him in Rhodesia, where until recently the condition was unknown. Another fact of great interest, and quoted by Dr. Sandwith, is that two cases of the disease have been recorded in persons who had never been outside the British Isles. This obviously bears on the question as to the method by which the parasite gains entrance to the body, and suggests the possibility of the disease being spread in some direct fashion from person to person. It seems impossible to believe that the stomach can be the avenue for invasion as the parasite cannot live in an acid medium. Possibly infection is effected through the skin, and in countries where the disease is endemic there is reason to think human beings may be attacked in the act of bathing. According to Mr. Mansell Moullin urotropin is of some value as a remedy, but Dr. Sandwith considers that this drug merely turns a septic into an ordinary cystitis and does not affect the number of ova in the urine; he thinks male fern to be more effective.

### Lord Rosebery, the County Council and Epsom.

It is not a little remarkable that Lord Rosebery, who claims to be the oldest and staunchest friend of the London County Council, should inferentially admit the extravagance of its late Progressive members, as illustrated by their purchase of the Horton Estate, at Epsom, and its utilisation. It appears that although the Long Grove and Manor Asylums provide for a population of 5,400 insane persons, together with a residential staff of quite one thousand, the County Council still has some 350 acres on the Horton Estate unappropriated for asylum purposes. The Asylums Committee properly pointed out to the Council that in their opinion the number of lunatic asylums on this estate was sufficient. This view was evidently based on the considerations that the site of an asylum should be readily accessible for patients and their friends, and that the present accommodation at Epsom exceeds the present requirements of the patients chargeable to unions on the south side of the Thames. They recommended therefore the purchase of a site on the North side, where there is a great deficiency in asylum accommodation, having regard to the 11,343 persons chargeable to unions on that side. The Finance Committee, however, on the ground of economy, opposed the purchase of further land and insisted on the erection of the new asylum at Epsom, despite its unsuitability for the reasons just shown. As Lord Rosebery urges, the 350 surplus acres on the Horton Estate might be sold to provide the purchase money for a site in the district where it is needed. The present position of matters illustrates the necessity that exists for Parliament to lay down a rule. It should provide a check through some central authority upon the overspending of public money by County Councils on the purchase of great sites not justified by the circumstances, and not suitable either, if any regard is to be had to the proper administration of our County Asylums in the best interests of the community and of those who supply the money. A tight hand should be brought to bear upon all expenditure of the kind, for the protection of the ratepayers and in the interests of the citizens. Lord Rosebery urges that it is a harsh stretch of power "to flood a country district with a mass of unhealthy, insanitary and mentally decayed persons." Still, our mental and other patients must be provided for, somewhere, though Epsom may have been harshly treated through the unchecked extravagance of a Progressive majority. The failure of Lord Rosebery's influence with the Progressives is a very remarkable fact.

